# Retrospective analysis of GnRH-a prolonged protocol for in vitro fertilization in 18,272 cycles in China

**DOI:** 10.1186/s13048-022-01044-7

**Published:** 2022-10-08

**Authors:** Lifeng Tian, Leizhen Xia, Qiongfang Wu

**Affiliations:** Center for Reproductive Medicine, Women’s and Children’s Hospital of Jiangxi Province, Nanchang, China

**Keywords:** Live birth rate, OHSS, In vitro fertilization, GnRH-a prolonged protocol, Oocyte retrieval

## Abstract

**Background:**

This large-cohort, retrospective study investigates the relationship between the number of oocytes retrieved and the clinical outcomes for patients receiving the GnRH-a prolonged protocol (mGnRH-a protocol) for fertilization in vitro or intracytoplasmic sperm injection–embryo transfer (IVF/ICSI-ET) treatment.

**Results:**

We categorized 18,272 cycles into three groups by the number of oocytes retrieved (1–8, 9–17, and ≥ 18) during IVF with the GnRH-a prolonged protocol at the Reproductive Medical Center of Jiangxi Maternal and Child Health Hospital from January 2014 to December 2018 (excluding oocyte donation cycles), analyzing the associations among oocyte number and live birth rates (LBRs) or cumulative LBRs (CLBRs), as well as the rate of moderate-to-severe ovarian hyperstimulation syndrome (OHSS). We defined the primary outcome as LBR and the secondary outcome to include the rate of patients at high risk for OHSS. The LBR (with fresh ET) per cycle of oocyte pick-up increased as the number of retrieved oocytes increased from 1 to ~ 8, plateaued between 9 ~ 17, and steadily decreased thereafter. However, the CLBR per cycle continued to increase as the oocyte number increased, as did the incidence of moderate-to-severe OHSS.

**Conclusions:**

Our results show a strong relationship between the number of oocytes retrieved and the CLBR following IVF treatment. The balance between treatment success and the risk of complications, especially OHSS, should be investigated further. We recommend a fresh-ET strategy for the GnRH-a prolonged protocol because the endometrial receptivity in the fresh cycles was better than those in the frozen cycles.

**Supplementary Information:**

The online version contains supplementary material available at 10.1186/s13048-022-01044-7.

## Background

Since the world’s first baby was born in 1978 using the IVF-ET technique developed by Dr. Robert Edwards and Dr. Patrick Steptoe [1], IVF-ET has been used widely for the treatment of infertility. In their initial successful procedure, these physicians collected oocytes during a natural ovulation period [2]. Normally, only one oocyte has the chance to mature and be fertilized in a natural menstrual cycle, with a low pregnancy rate. Controlled ovarian stimulation (COS) can cause multiple follicle development in a single cycle [3], producing more mature oocytes and available embryos [4], as well as significantly improving regulation of the pregnancy rate.

Ovarian stimulation is an important part of assisted reproduction treatment. COS can induce the development of multiple follicles, facilitating the retrieval of oocytes and thereby enabling optimization of the pregnancy rate. Gonadotropin-releasing hormone agonist (GnRH-a) treatment is an important component of controlled ovarian stimulation protocols for many patients. Since its development, GnRH-a treatment has increased patients’ retrieved oocyte numbers and pregnancy rates and reduced the number of cycle cancelations. During the past 40 years, many COS protocols have been developed, such as the long gonadotropin-releasing hormone agonist (GnRH-a) protocol, the GnRH-a prolonged protocol, the GnRH antagonist (GnRH-ant) protocol, the mild stimulation protocol, and the luteal-phase ovulation stimulation protocol.

The optimal yield from COS can range from 6 to 15 oocytes [4–10]. A low oocyte yield limits the production of high-quality embryos, which can affect the pregnancy rate, whereas a high oocyte yield may be accompanied by overproduction of estradiol (E2) and severe ovarian hyperstimulation syndrome (OHSS), affecting endometrial receptivity.

For young women with a normal ovarian reserve in a long GnRH-a protocol, retrieving 10∼12 oocytes might result in optimized pregnancy outcomes in a fresh-ET cycle with low OHSS risk and would not compromise cumulative outcomes. When ≥ 16 oocytes are retrieved, a “freeze-all” embryo strategy might be preferable [11]. However, a prospective study by Fatemi et al. has shown that a high ovarian response rate (> 18 oocytes) did not compromise the chance of ongoing pregnancy following fresh ET and even increased the chance of cumulative ongoing pregnancy in a GnRH-ant protocol [5].

Many studies have examined the optimal oocyte number for the conventional long GnRH-a protocol and the GnRH-ant protocol, but fewer study has examined the optimal oocyte number for the GnRH-a prolonged protocol. The GnRH-a prolonged protocol also had been known as the early-follicular-phase long-acting GnRH-a long (EFLL) protocol, and it was initially applied in a Chinese in vitro fertilization (IVF) center. In recent years, it has become the mainstream protocol in most reproductive medicine centers in China, originally started from our center, due to its enhancement of endometrial receptivity, the pelvic microenvironment, embryo implantation and clinical pregnancy rates and its reduction of the abortion rate in the normal patient population. In addition, the optimal number of oocytes has not yet been determined unequivocally and can vary by COS protocol. Cheon et al. have suggested that the GnRH-a prolonged protocol is a useful alternative for improving patient convenience with their clinical outcomes as compared to the conventional long GnRH-a protocol in controlled ovarian hyperstimulation (COH) for IVF-ET cycles [12]. This COS protocol is the most widely used and has been associated with the best pregnancy outcomes at our center (the clinical pregnancy rate has been stable at over 60% since 2009) [13–20]. Therefore, in this retrospective study, we explore the associations among the optimal number of oocytes retrieved and the live birth rate (LBR), cumulative LBR (CLBR), and incidence of OHSS for this protocol.

## Results

### Demographic and IVF/ICSI data

We recruited 17,637 patients with 18,272 cycles receiving GnRH-a prolonged protocol and IVF/ICSI treatment during the study period (2014–2018). Table 1 summarizes patient demographics and infertility and cycle characteristics.

**Table 1:** Characteristics of 18,272 cycles and IVF/ICSI cycles

Characteristics	Values
Maternal age (years), *n* (%)	
18–34	14,316 (78.35%)
35–37	2,057 (11.26%)
38–39	1,041 (5.7%)
≥ 40	858 (4.7%)
Number of previous IVF cycles, *n* (%)	
0	15,616 (85.46%)
1	1,694 (9.27%)
2	610 (3.34%)
≥ 3	352 (1.93%)
Duration of infertility (years), (x̄ ± SD)	4.50 ± 3.23
Type of infertility, *n* (%)	
Primary	8,053 44.10%)
Secondary	10,219 (55.90%)
Cause of infertility, *n* (%)	
Tube disease	13,332 (72.96%)
Male factor	5,109 (27.96%)
Endometriosis	1,234 (6.75%)
Anovulation	2,761 (15.11%)
Insemination method, *n* (%)	
IVF	13,826 (75.86%)
ICSI	3,452 (18.94%)
IVF + ICSI	948 (5.20%)
No. oocytes retrieved	
Median [IQR]	12 [816]
Year beginning IVF cycle, *n* (%)	
2014	2,027 (11.09%)
2015	2,953 (16.16%)
2016	4,297 (23.52%)
2017	4,656 (25.48%)
2018	4,339(23.75%)
No. transferable embryos	
Median [IQR]	3 [2,3,4]
AFC, Median [IQR]	12 [9,17]
BMI, kg/m^2^, (x̄ ± SD)	21.87 ± 3.05
Basal FSH (IU/L), Median [IQR]	6.305 [5.4, 7.43]
Basal E2 (pg/mL), Median [IQR]	35.8 [26.6, 47.84]
Basal LH (IU/L), Median [IQR]	4.5 [3.31, 6.18]

*ICSI* Intracytoplasmic sperm injection, *IVF* In vitro fertilization, *AFC* Antral follicle count, *BMI* Body mass index, *FSH* Follicle-stimulating hormone, *E2* Estradiol, *LH* Luteinizing hormone

### Number of oocytes retrieved is associated with LBR, CLBR, and OHSS

**Fig. 1:**
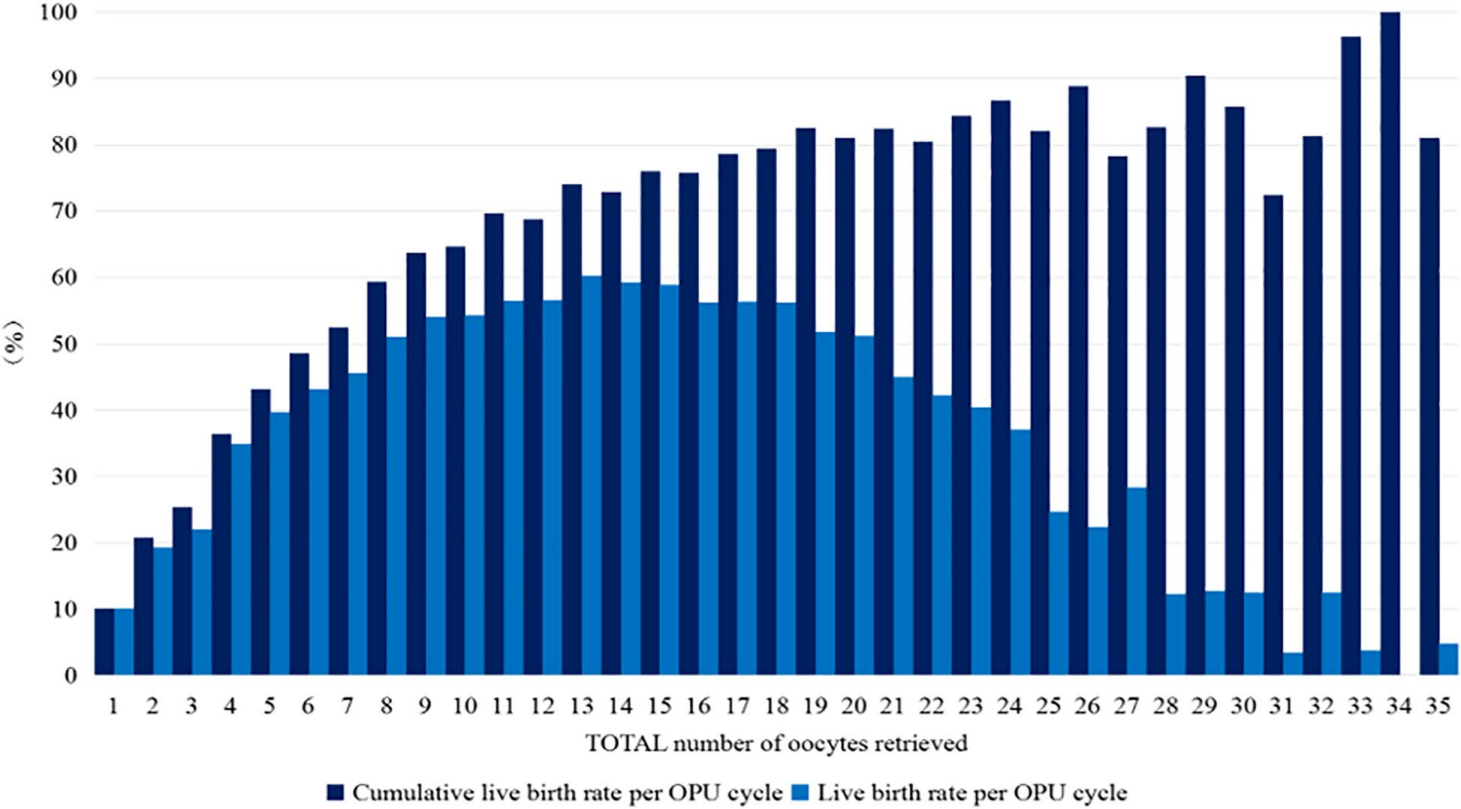
LBRs in relation to the number of oocytes retrieved. *LBRs* live birth rates

**Fig. 2:**
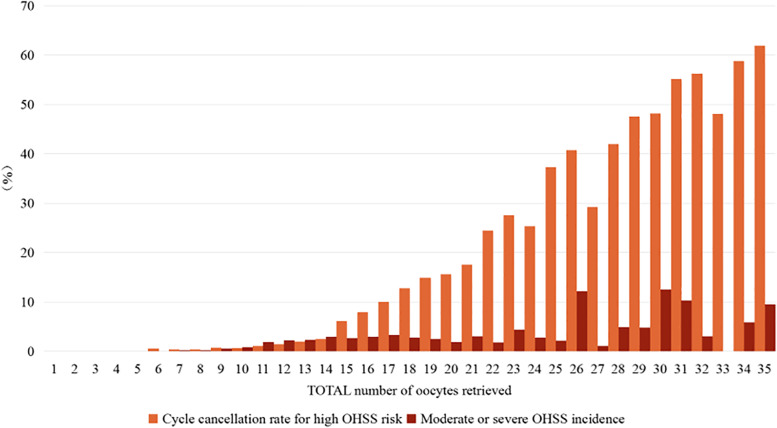
Rates of OHSS and cycle cancellation in relation to the number of oocytes retrieved. *OHSS* Ovarian hyperstimulation syndrome

We plotted the LBR and CLBR per OPU cycle against the number of oocytes retrieved (Fig. 1, Supplementary Fig. 1), as well as the cancellation rate for high OHSS risk and the incidence of moderate-to-severe OHSS (Fig. 2, Supplement Fig. 2). As the number of oocytes increased, the fresh-ET LBR per OPU cycle initially rose to retrieval of ~ 8 oocytes, to a plateau between 9 ~ 17 oocytes, and decreased with ≥ 18 oocytes (Fig. 1). By contrast, the rate of cycle cancellation for the high risk of OHSS began to increase notably at > 15 oocytes and continued to increase up to the highest level of oocyte retrieval (Fig. 2). The incidence of moderate-to-severe OHSS increased as the number of retrieved oocytes increased (Fig. 2). The CLBR was also increasing as the number of retrieved oocytes increased and was higher than the LBR at all points (Fig. 1).

### Patient characteristics and IVF outcomes vary significantly by the number of retrieved oocytes

Using the curve of the fresh-ET LBR per OPU cycle under each oocyte number, we divided the patients into three groups (≤ 8, 9 ~ 17, and ≥ 18 retrieved oocytes) and determined and compared their clinic outcomes and demographic data (Table 2). We observed significant differences among the group values of the continuous variables and among the distributions of the categorical variables for age, BMI, duration of ovarian stimulation, levels of P and E2 on hCG trigger day, number of available embryos, time to live birth, percentage of participants with all embryos frozen (for patients with a high OHSS risk), moderate-to-severe OHSS rate, and CLBR (which had positive associations with oocyte numbers; all *P* < 0.0001). The AFC, total dose of Gn, LH level on the hCG trigger day, number of fresh embryos transferred, and cancellation rate all had negative associations with the oocyte number (all *P* < 0.0001). The fresh-ET LBR per OPU cycle was higher in Group 2 than in Groups 1 and 3.

**Table 2:** Patient demographics and IVF outcomes, by number of retrieved oocytes

	Group 1	Group 2	Group 3	F (ANOVA) or *χ*^*2*^ test statistic	*P*-value
	≤ 8 oocytes	9–17 oocytes	≥ 18 oocytes		
No. treatment cycles (*n*)	4870	10,106	3296		
Age (years), x̄ ± SD	32.40 ± 5.20	30.09 ± 4.69	28.48 ± 4.16	724.77	< 0.0001
BMI (kg/m^2^), x̄ ± SD	22.06 ± 3.11	21.83 ± 3.02	21.74 ± 3.07	12.36	< 0.0001
AFC, Median [IQR]	9 [6,12]	13 [10,17]	16 [12,20]	2252.7981	< 0.0001
Total dose of gonadotropins (IU), Median [IQR]	2,700 [2,025, 3,525]	2,100 [1,537.5, 2,775]	1,762.5 [1,350, 2,475]	1685.9298	< 0.0001
Duration of ovarian stimulation (days), x̄ ± SD	11.35 ± 2.21	11.70 ± 2.10	12.22 ± 2.46	153	< 0.0001
LH on hCG trigger day (IU/L), Median [IQR]	0.93 [0.6,1.4]	0.88 [0.57,1.36]	0.79 [0.49,1.23]	107.741	< 0.0001
P on hCG trigger day (ng/mL), Median [IQR]	0.658 [0.43, 0.89]	0.82 [0.58, 1.07]	0.98 [0.711, 1.28]	1272.15	< 0.0001
E2 on hCG trigger day (pg/mL), Median [IQR]	1,214 [879.1, 1,670]	2,184 [1,635, 2,913]	3,478 [2,602.22, 4,608.5]	6.943.40	< 0.0001
Endometrial thickness on hCG trigger day (mm), Median [IQR]	10.6 [9, 12.4]	10.9 [9.4, 12.6]	10.8 [9.2, 12.5]	51.1977	< 0.0001
Oocytes retrieved, x̄ ± SD	6.05 ± 1.79	12.53 ± 2.47	22.27 ± 5.01	29,589.6	< 0.0001
No. available embryos, Median [IQR]	2 [1 3]	3[2,4]	5 [3,7]	3742.67	< 0.0001
No. fresh embryos transferred, x̄ ± SD	1.80 ± 0.41	1.86 ± 0.35	1.64 ± 0.48	258.05	< 0.0001
Time to live birth (days), x̄ ± SD	320.96 ± 98.26	330.08 ± 266.13	383.77 ± 187.53	65.79	< 0.0001
Cancellation rate with no available embryos, *n* (%)	627/4,870 (12.87%)	458/10,106 (4.53%)	80/3,296 (2.43%)	488.223	< 0.0001
Cycle cancellation for high risk of OHSS, *n* (%)	14/4,870 (0.29%)	305/10,106 (3.02%)	782/3,296 (23.73%)	2268.21	< 0.0001
Moderate-to-severe OHSS rate, *n* (%)	4/4,870 (0.08%)	211/10,106 (2.09%)	112/3,296 (3.4%)	134.413	< 0.0001
CLBR/cycle started, *n* (%)	2,314/4,870 (47.52%)	7,143/10,106 (70.68%)	2,717/3,296 (82.43%)	1244.99	< 0.0001
No. fresh transfer cycles (*n*)	4057	8878	1922		
No. embryos transferred				504.44	< 0.0001
1	826/4057(20.36%)	1240/8878(13.97%)	685/1922(35.64%)		
2	3231/4057(79.64%)	7638/8878(86.03%)	1237/1922(64.36%)		
Embryo transfer type				1610.83	< 0.0001
Cleavage embryo	3933/4057(96.94%)	8296/8878(93.44%)	1283/1922(66.75%)		
Blastocyst	124/4057(3.06%)	582/8878(6.56%)	639/1922(33.25%)		
HCG positive rate	2713/4057(66.87%)	6889/8878(77.6%)	1561/1922(81.22%)	215.12	< 0.0001
Clinical pregnancy rate	2431/4057(59.92%)	6378/8878(71.84%)	1470/1922(76.48%)	240.7	< 0.0001
Abortion rate	347/2431(14.27%)	557/6378(8.73%)	92/1470(6.26%)	84.83	< 0.0001
Live birth rate	2044/4057(50.38%)	5743/8878(64.69%)	1361/1922(70.81%)	320.48	< 0.0001

The CLBR corresponded to the results of all treatments from 1 complete cycle, including all fresh and frozen–thawed ET cycles from 1 oocyte retrieval, over a time period of 2 years

Available embryos, high-quality embryos for transfering

*AFC* Antral follicle count, *ANOVA* Analysis of variance, *BMI* Body mass index, *CLBR* Cumulative live birth rate, *E2* Estradiol, *hCG* human choriogonadotropin, *IVF* In vitro fertilization, *LBR* Live birth rate, *LH* Luteinizing hormone, *OHSS* Ovarian hyperstimulation syndrome, *OPU* Oocyte pick-up, *P* Progesterone

### Multivariable analysis of variables associated with CLBR and OHSS

We tested the variables for association with CLBR and OHSS using multivariable logistic regression analyses, the results of which are shown in Tables 3 and 4. In the analysis of oocyte number as a variable, Group 1 was the reference group, and the adjusted odds ratios (ORs) for CLBR were 2.07 (1.89–2.26) in Group 2 and 3.15 (2.75–3.62) in Group 3 (*P* < 0.0001) (Table 3). Age, BMI, AFC, number of previous treatment cycles per patient, and duration of infertility were all significantly associated with CLBR in the multivariable model (Table 3). In the analysis of the cause of infertility as a variable, we used unexplained infertility as the reference group; only anovulatory and malefactors had a positive association with CLBR, whereas endometriosis had a negative association. However, for the adjusted OR, only endometriosis had a negative association (*P* = 0.0036).

**Table 3:** Multivariable analysis of the association of the variables with CLBR

Independent covariates	Covariate strata	Crude OR (95% CI)	*P*-value	Adjusted OR (95% CI)	*P*-value
Age		0.90 (0.89–0.90)	< 0.0001	0.93 (0.92–0.94)	< 0.0001
BMI		0.98 (0.97–0.99)	< 0.0001	0.98 (0.97–1.00)	0.0062
No. retrieved oocytes					
	Group 1 to Group2	2.66 (2.48–2.86)	< 0.0001	2.07 (1.89–2.26)	0.0003
	Group1 to Group 3	5.18 (4.66–5.76)	< 0.0001	3.15 (2.75–3.62)	< 0.0001
AFC		1.08 (1.07–1.08)	< 0.0001	1.03 (1.02–1.03)	< 0.0001
No. previous treatment cycles/patient		0.72 (0.69–0.76)	< 0.0001	0.79 (0.73–0.86)	< 0.0001
Duration of infertility		0.94 (0.93–0.95)	< 0.0001	0.98 (0.97–0.99)	0.0002
Cause of infertility					
	Unexplained	1		1	
	Tubal	1.01(0.94–1.08)	0.8696	0.92 (0.84–1.01)	0.0856
	Endometriosis	1.20 (1.06–1.35)	0.0032	1.26 (1.08–1.47)	0.0036
	Anovulatory	0.66 (0.60–0.73)	< 0.0001	1.13 (0.99–1.28)	0.0706
	Male factor	0.92 (0.86–0.98)	0.0158	1.03 (0.94–1.12)	0.5714

*P*-values correspond to differences determined by Cox regression analyses with CLBR as the outcome (dependent) variable

*AFC* Antral follicle count, *BMI* Body mass index, *CI* Confidence interval, *CLRB* Cumulative live birth rate, *FSH* Follicle-stimulating hormone

**Table 4:** Multivariable analysis of the association of the variables with moderate-to-severe OHSS

Independent covariates	Covariate strata	Crude OR (95% CI)	*P*-value	Adjusted OR (95% CI)	*P*-value
Age		0.93 (0.91–0.96)	< 0.0001	0.99 (0.96–1.02)	0.5496
BMI		0.92 (0.88–0.96)	< 0.0001	0.91 (0.87–0.95)	< 0.0001
AFC		1.10 (1.08–1.11)	< 0.0001	1.08 (1.06–1.10)	< 0.0001
No. retrieved oocytes					
	Group 1 to Group 2	25.94 (9.64–69.8)	< 0.0001	15.06 (5.57–40.76)	< 0.0001
	Group 1 to Group 3	42.79 (15.77–116.12)	< 0.0001	21.24 (7.65–58.96)	< 0.0001
ET strategy					
	All embryos frozen	1		1	
	1 D3 embryo transferred	0.29 (0.14–0.57)	0.0012	0.60 (0.28–1.29)	0.1020
	1 blastocyst (Day 5/6 embryo) transferred	1.08 (0.70–1.67)	0.1246	0.82 (0.51–1.32)	0.3396
	2 D3 embryos transferred	0.85 (0.65–1.11)	0.5886	1.32 (0.95–1.83)	0.1812
	2 blastocysts transferred	1.04 (0.25–4.32)	0.5998	1.78 (0.42–7.55)	0.3529

*P*-values correspond to differences determined by Cox regression analyses, with moderate-to-severe OHSS as the outcome (dependent) variable

*AFC* Antral follicle count, *BMI* Body mass index, *ET* Embryo transfer, *CI* Confidence interval, *OHSS* Ovarian hyperstimulation syndrome

For the associations with moderate-to-severe OHSS, in the analysis of oocyte number as a variable, Group 1 was the reference group, and the adjusted ORs were 15.06 (5.57–40.76) in Group 2 and 21.24 (7.65–58.96) in Group 3 (*P* < 0.0001) (Table 4). In the analysis of the ET strategy as a variable, individuals with all embryos frozen (no fresh ET) made up the reference group, and the adjusted ORs were 0.60 (0.28–1.29) in the group receiving 1 Day 3 embryo, 0.82 (0.51–1.32) in the group receiving 1 blastocyst (Day 5/6 embryo), 1.32 (0.95–1.83) in the group receiving 2 Day 3 embryos, and 1.78 (0.42–7.55) in the group receiving 2 blastocysts. The risk of moderate-to-severe OHSS increased with the number of ETs and embryo phase; however, none of the ET strategies showed a significant difference. BMI and AFC (but not age) were also significantly associated with moderate-to-severe OHSS.

## Discussion

The question of how to define a successful IVF has not yet been answered unequivocally. For infertility treatment, the outcomes of the natural cycle or single-follicular development cycle are unsatisfactory. An important step toward the achievement of optimal pregnancy outcomes has occurred with the development of COS, which induces multiple follicular developments, thus enabling the transfer of the best embryos derived from harvested oocytes, with cryopreservation of any surplus, high-quality embryos.

The success of IVF regimens is measured by the numbers of oocytes and embryos and by pregnancy rates. However, the number of oocytes does not necessarily correlate with the number of high-quality embryos. Previous studies have shown that gonadotropin dosage is negatively correlated with fertilization rates and with the rate of blastocyst formation in mice. High doses of Gn can negatively affect the developmental potential of mouse embryos but do not affect the cleavage rate of these embryos. Appropriate administration of Gn can enable the production of a satisfactory number of oocytes, with the benefit of optimizing the developmental potential of the resultant embryos [21]. Notably, overproduction of oocytes is accompanied by high levels of estrogen and corresponding incidence of OHSS, along with the reduction of endometrial receptivity, which can affect embryo implantation. Therefore, the number of oocytes retrieved during IVF is related to the incidence of OHSS, as well as to treatment cost and pregnancy outcomes.

Mild ovarian stimulation has emerged as a safer method of IVF compared with conventional stimulation IVF (C-IVF), with regards to reducing the risk of OHSS and treatment-related stress. But there had not been a strand optimal number of retrieved oocytes in the clinical practice. A previous study of 400,135 IVF cycles of the UK suggested 15 was the optimal number of retrieved oocytes [8]. And the result from 2,226 patients of US suggested that the pregnancy rate was higher when the retrieved oocytes number was ≥ 15 [22]. A recent study of 8676 cycles of first fresh embryo transfer in the Chinese population detected that the optimal ovarian response for retrieved oocytes was ≥ 10 [23]. Our results suggest that an oocyte yield of 9 ~ 17 is ideal, giving the highest LBR per OPU cycle (54.0%-60.3%), as well as moderating the risk of OHSS. When ≥ 18 oocytes were retrieved, the rate of cycle cancellation because of the high risk of OHSS significantly increased to 12.8%. Notably, the fresh ET also can minimize the time from treatment to living birth, with our results showing that the time to live birth increased with the number of oocytes retrieved, especially when ≥ 18 oocytes were collected. Because the greatest risk of patients dropping out of IVF treatment occurs after the first cycle [24, 25], fresh ET is an important strategy.

The GnRH-a long protocol remains the most frequently used COS protocol in IVF treatment [26]. Many studies have focused on the optimum number of oocytes retrieved in the classic GnRH-a long protocol [9, 11, 27], but no relevant results exist for the early follicular phase with the GnRH-a prolonged protocol.

Cheon et al. and Ying et al. have found that the prolonged protocol (a single administration of long-acting GnRH-a at 3.75 mg) can improve patient convenience with clinical outcomes due to its better endometrial receptivity, as compared with daily administrations of short-acting GnRH-a per fresh-ET cycle [12, 28]. The prolonged protocol has been used increasingly at several IVF centers in China, and evidence has suggested that the LBR may be higher when using the prolonged protocol than when using the long protocol [29]. In the past seven years, the GnRH-a prolonged protocol has been widely used in our center for patients with different causes of infertility, accounting for 60%–70% of treatments each year, for a fresh-ET rate per stimulation cycle of over 70%, and for a high clinical pregnancy rate of 68% in 2015 [14, 15, 17, 20]. The prolonged protocol has a slightly longer stimulation time and results in lower levels of E2 and P on the hCG trigger day than does the long protocol. Because of its higher pregnancy rate, convenience, and lower cancellation rate, to find the best clinical outcomes under this protocol, the study of the optimal number of oocytes retrieved in the prolonged protocol can guide clinical work.

In this study, multivariable analyses showed that CLBR was negatively associated with age, BMI, the number of previous IVF treatments, duration of infertility, and endometriosis but was positively associated with the number of retrieved oocytes and AFC. We evaluated the ovarian response and generated a treatment protocol using patient age, BMI, AFC, duration and cause of infertility, and previous treatment history. The multivariable analysis also showed that moderate-to-severe OHSS was positively associated with AFC and the number of retrieved oocytes but negatively associated with BMI. To maximize the LBR from the fresh-ET cycle, we recommend that the optimal number of oocytes should be between 9 ~ 17. However, when ≥ 15 oocytes are retrieved, one must carefully assess the risk of OHSS, perhaps using a single-ET strategy to avoid OHSS, and, if necessary, adopting a freeze-all strategy.

The number of oocytes retrieved following COS has a strong association with clinical outcomes, so it is important to determine how to control this number. Regulatory strategies mainly include the use of individualized protocols for COS, re-evaluation of the ovarian reserve before COS, and adjustment of the COS process. Individualized treatment in IVF should be based on a prediction of the patient’s ovarian response and parameters such as age, medical treatment history, AMH levels, AFC, basic FSH levels, and previous COS history [13, 30]. These data can help identify whether a woman is likely to have a normal, poor, or hyperactive ovarian response so that the appropriate treatment protocol can be chosen.

Our study had three important limitations. First, generalizability is limited by the nature of the patient population. The proportion of young patients (18–34 years, 78.35%) and first-cycle patients (85.46%) in our center was high, and the average BMI was low (21.87 ± 3.05 kg/m^2^). Second, all patients in our study were treated at a single reproductive medical center with the same prolonged COS protocol. In addition, the sample size was not large enough to reach a reliable conclusion. Therefore, further prospective analyses and multicenter studies with larger sample sizes and different protocols are warranted.

## Conclusions

Our results show a strong relationship between the number of oocytes retrieved and the CLBR following IVF treatment. It is important to determine how to optimize the number of oocytes produced by COS. Regulation strategies mainly include the formulation of individualized COS protocols, the reassessment of ovarian function before COS, and regulation during the COS process. Based on our findings, we recommend a fresh-ET strategy for the GnRH-a prolonged protocol, because the endometrial receptivity in the fresh cycles was better than those in the frozen cycles. The optimal number of oocytes for achieving the best chance of live birth in the first IVF cycle and for higher chances of live birth in cumulative cycles is 9 ~ 17. The optimal number of oocytes can vary by protocol, but because patient safety and health are the most important factors to consider, the risk of OHSS should be evaluated carefully and minimized.

## Methods

### Patients

We reviewed the medical records for patients who underwent IVF/intracytoplasmic sperm injection (ICSI)-ET treatment between January 2014 and December 2018 in the Reproductive Medical Center of Jiangxi Provincial Maternal and Child Health Hospital. The inclusion criterion was IVF/ICSI-ET treatment with the GnRH-a prolonged protocol; the exclusion criteria were cycle cancellation before oocyte pick-up (OPU); lack of oocyte retrieval after OPU; oocyte donation, sharing, and cryopreservation; frozen oocyte thawing; and preimplantation genetic testing. We excluded patients with the following current conditions: uncontrolled diabetes; hepatic or renal dysfunction without a definite clinical diagnosis; history of deep-vein thrombosis; history of pulmonary embolism; history of cerebrovascular events; uncontrolled hypertension; heart disease; suspicion of cervical, endometrial, or breast cancer; or unexplained vaginal bleeding.

We collected data from the clinical records for the following demographic and clinical characteristics: age; body mass index (BMI); antral follicle count (AFC); duration, type, and cause of infertility; basic hormone levels; Gn dose; days of ovarian stimulation; the number of oocytes retrieved; type of insemination; two-pronuclear zygote fertilization rate; the number of embryos transferred in the fresh-ET cycle; the number of transferable embryos; rate of moderate-to-severe OHSS; cycle cancellation rate; embryo implantation and abortion rates; and LBR and CLBR. The primary outcome was the number of oocytes retrieved.

### Treatment protocol

#### GnRH-a prlonged and embryo freezing protocol

We performed COS using an GnRH-a prlonged protocol. Patients received a single dose s.c. injection of 3.75 mg GnRH-a (long-term-acting disheveling; Beaufour Ipsen, Dreux, France) on Day 2 ~ 3 of the cycle, after the ultrasound scan confirmed ovarian quiescence and the presence of a thin endometrium (< 5 mm). When complete pituitary desensitization was achieved (28 days after the initiation of GnRH-a), with a low plasma E2 level of ≤ 30 pg/ml and an LH level of ≤ 2 IU/l, COS was started. For every individual, we selected the dosage of stimulating Gn based on age, AFC, basal FSH, BMI, and previous ovarian response [30, 31]. During stimulation, we monitored the ovarian response through assessments of serum E2, progesterone (P), and LH, as well as serial transvaginal ultrasonographic examinations. We would adjust the Gn doses when needed.

On identification of at least one follicle with a diameter ≥ 19 mm or two follicles with diameters ≥ 18 mm, we administered 250 μg of recombinant human choriogonadotropin (hCG [Ovitrelle]; Merck Serono, Corsier-sur-Vevey, Switzerland) subcutaneously. We performed oocyte retrieval 36 h after injection of hCG using a transvaginal ultrasonography-guided puncture of the follicles. Semen was produced by masturbation, and motile spermatozoa were prepared by density gradient centrifugation and the swim-up procedure. We initiated luteal support after OPU using intramuscular injection of P (80 mg/day). Type of insemination included IVF, ICSI, and early-rescue ICSI. All the oocytes were inseminated 4–5 h after collection, fertilization was initially assessed 5 h after IVF insemination, and if the oocytes had not been fertilized at this point, early-rescue ICSI was performed immediately.

We selected the highest quality embryos, consisting of 7–9 blastomeres of uniform size and with a fragment proportion < 20% [32], for embryo transfer or cryopreservation on Day 3 after fertilization. We evaluated individuals with ≥ 15 retrieved oocytes on the day of embryo transfer for ovarian diameter ≥ 7 cm and/or reported abdominal distension or bloating, which are indications for embryo cryopreservation, to avoid moderate-to-severe OHSS. All these embryos were cryopreserved by vitrification using the Cryotop system [33].

#### The preparation of the endometrium

All FET cycle individuals were divided into three groups, the natural cycle, the HRT cycle, and the GnRHa-HRT cycle, for the preparation of the endometrium. The natural cycle was suitable for individuals who have regular menstrual cycles and can ovulate normally. According to the length of the patient's menstrual cycle (21–35 days), the follicle and endometrium are monitored by B-ultrasonography from the middle follicular phase. When the diameter of the follicle reaches 14-15 mm, the B-ultrasonography and serum LH and E2 levels are monitored every day until the day of ovulation. The natural cycle recommends LH peak + 4d (D3 cleavage-stage embryo) or LH peak + 6d (D5 blastocyst) as the timing of embryo transfer. To improve the natural cycle, when the diameter of the dominant follicle is more than 16 mm and the intima thickness exceeds 7-8 mm, hCG can be used clinically to replace the endogenous LH peak to induce ovulation, and then arrange the timing of embryo transfer. It is recommended that hCG injection day + 5 days (D3 cleavage stage embryos) or HCG injection day + 7 days (D5 blastocysts) as the timing of embryo transfer. The HRT cycle was suitable for individuals with ovulation disorders or irregular menstruation. It can also be used for patients with regular menstruation but periodic monitoring of anovulation, or patients who are inconvenient for frequent trips to the hospital to monitor ovulation. The Estrogen was begun to use at 2–3 days later of menstrual cramps. The estrogen administration route of administration includes oral, vaginal suppository, and transdermal absorption. A fixed regimen (oral dose 6 mg/d) or incremental regimen (usually 1–4 days, 4 mg/d; 5–8 days, 6 mg/d; 9th day, monitor the endometrial thickness, if > 7 mm, maintain 6 mg/d, if < 7 mm, increase the amount to 8 mg/d) can be used. The GnRHa-HRT cycle was used for endometriosis, adenomyosis, thin endometrium, unexplained repeated implantation failure, polycystic ovary syndrome, pelvic surgery history, or menstrual high progesterone. GnRH-a (3.75 mg) was used on individuals every 28 days starting on the 2-3th day of menstruation. According to the individuals’ specific situation can be injected 1–6 times, 28 days after the last injection to review endocrine hormone levels and transvaginal B-ultrasonography, blood hormone levels reached the standard after entering the cycle, estrogen supplement with HRT cycle.

#### Embryos transfer

**Fig. 3:**
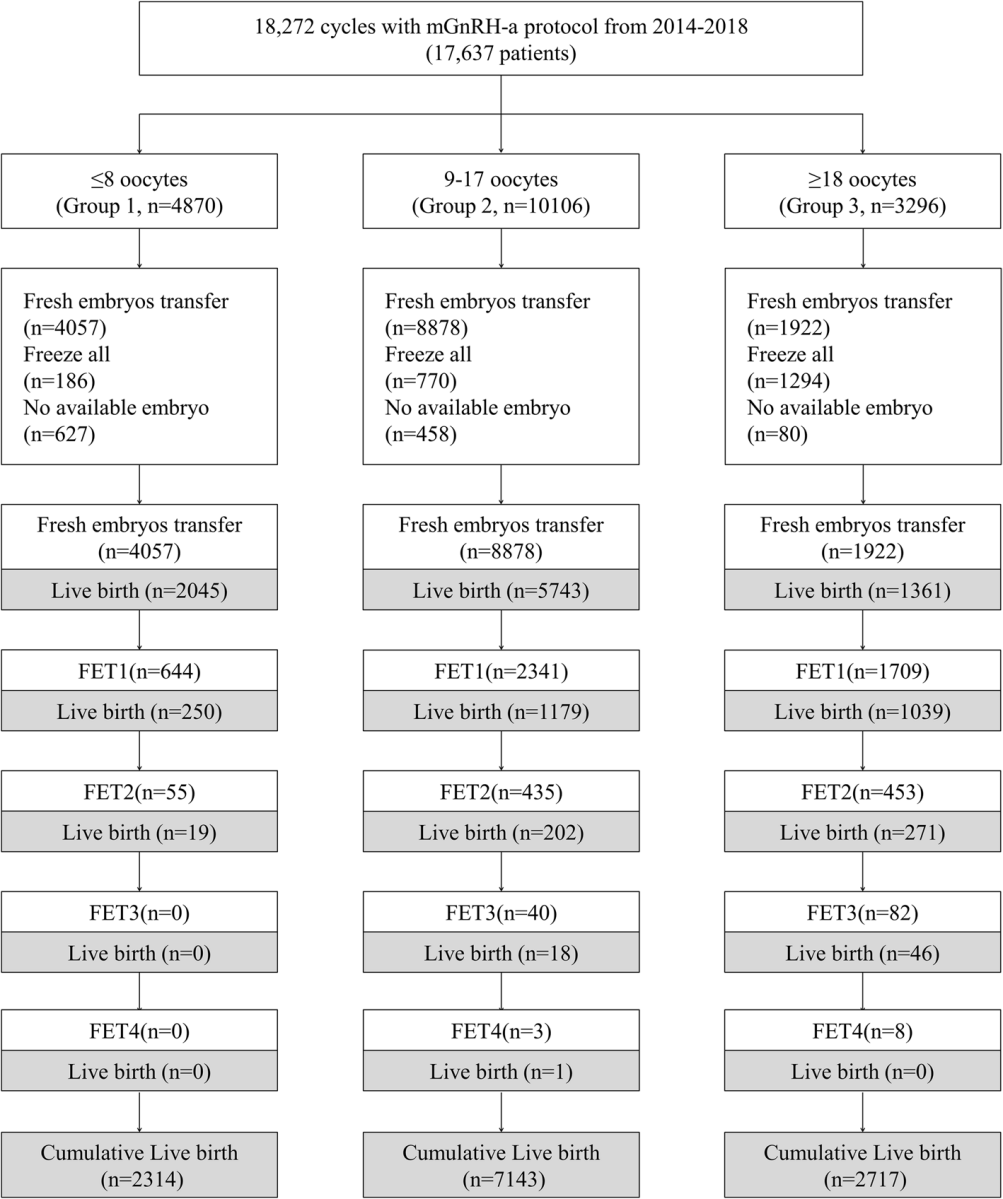
The flow chart of the consecutive analysis steps

The number of embryos transferred (≤ 2 per patient) complied with the national regulations in China and conformed to individual patient requests. We evaluated individuals with ≥ 15 retrieved oocytes on the day of embryo transfer for ovarian diameter ≥ 7 cm and/or reported abdominal distension or bloating, which are indications for embryo cryopreservation, to avoid moderate-to-severe OHSS. In the few patients who had indications for blastocyst transfer [34], we performed embryo transfer on Day 5. We categorized blastocyst quality as excellent (AA), good (AB, BA, BB), fair (BC, CB), or poor (CC) based on trophectoderm and inner-cell-mass quality scores [35]. We supported the luteal phase through the daily intravaginal administration of 90 mg of P gel (Crinone gel 8%; Merck Serono) and of 20 mg dydrogesterone (Duphaston 10 mg/tablet; Solvay Pharma, Weesp, Netherlands) after embryo transfer. We assessed reproductive outcome 2 weeks after embryo transfer testing for hCG; then at gestational Weeks 7–9, when a positive assessment was deemed a clinical pregnancy; and finally at delivery, when the measured outcome was live birth. We defined positive hCG as plasma hCG > 5 IU/L and clinical pregnancy as detection of a gestational sac and a heartbeat, verifying a living fetus using ultrasonography. We defined live birth as at least one living child from the fresh ET, irrespective of the duration of gestation. The CLBR corresponded to the results of all treatments from one complete cycle, including all fresh and frozen-thawed ET cycles from one oocyte retrieval, over 2 years. The follow-up period was 2 years. We maintained luteal support until 10 weeks of pregnancy. We recorded pregnancy complications, as well as neonatal birth weight and complications at delivery. A flow chart of the consecutive analysis steps is depicted in Fig. 3.

### Statistical analysis

We analyzed the data using the statistical software SAS, version 9.4 (SAS Institute, Cary, NC, USA). We compared continuous variables using analysis of variance (ANOVA), summarizing them as mean ± standard deviation (x̄ ± SD). We summarized the data that did not fit a normal distribution by the median (interquartile range [IQR]). We determined the count data adoption rate (%) using a chi-square test. We used logistic regression for multivariate analysis, setting the test level α to 0.05 and considering *P*-values < 0.05 as statistically significant.

## Supplementary Information

**Additional file 1: Supplementary Figure 1.** LBRs in relation to the number of oocytes retrieved. A: <35years-old; B: 35~37 years-old; C: >38 years-old. **Supplementary Figure 2.** Rates of OHSS and cycle cancellation in relation to the number ofoocytes retrieved. A: <35 years-old; B: 35~37 years-old; C: >38 years-old.

## Data Availability

All data generated through this study are included in this article.

## Abbreviations

GnRH-a: Gonadotropin-releasing hormone agonist
OHSS: Ovarian hyperstimulation syndrome
COS: Controlled ovarian stimulation
COH: Controlled ovarian hyperstimulation
LBR: Live birth rate
CLBR: Cumulative LBR
