# Supplementary material for: Retrospective analysis of GnRH-a prolonged protocol for in vitro fertilization in 18,272 cycles in China
Source: J Ovarian Res. 2022 Oct 8;15:110. doi: 10.1186/s13048-022-01044-7 (PMC9548105; doi:10.1186/s13048-022-01044-7)
Supplement: Supplementary file 1 — Additional file 1: Supplementary Figure 1. LBRs in relation to the number of oocytes retrieved. A: <35years-old; B: 35~37 years-old; C: >38 years-old. Supplementary Figure 2. Rates of OHSS and cycle cancellation in relation to the number ofoocytes retrieved. A: <35 years-old; B: 35~37 years-old; C: >38 years-old. [file 13048_2022_1044_MOESM1_ESM.pptx]

## Slide 1
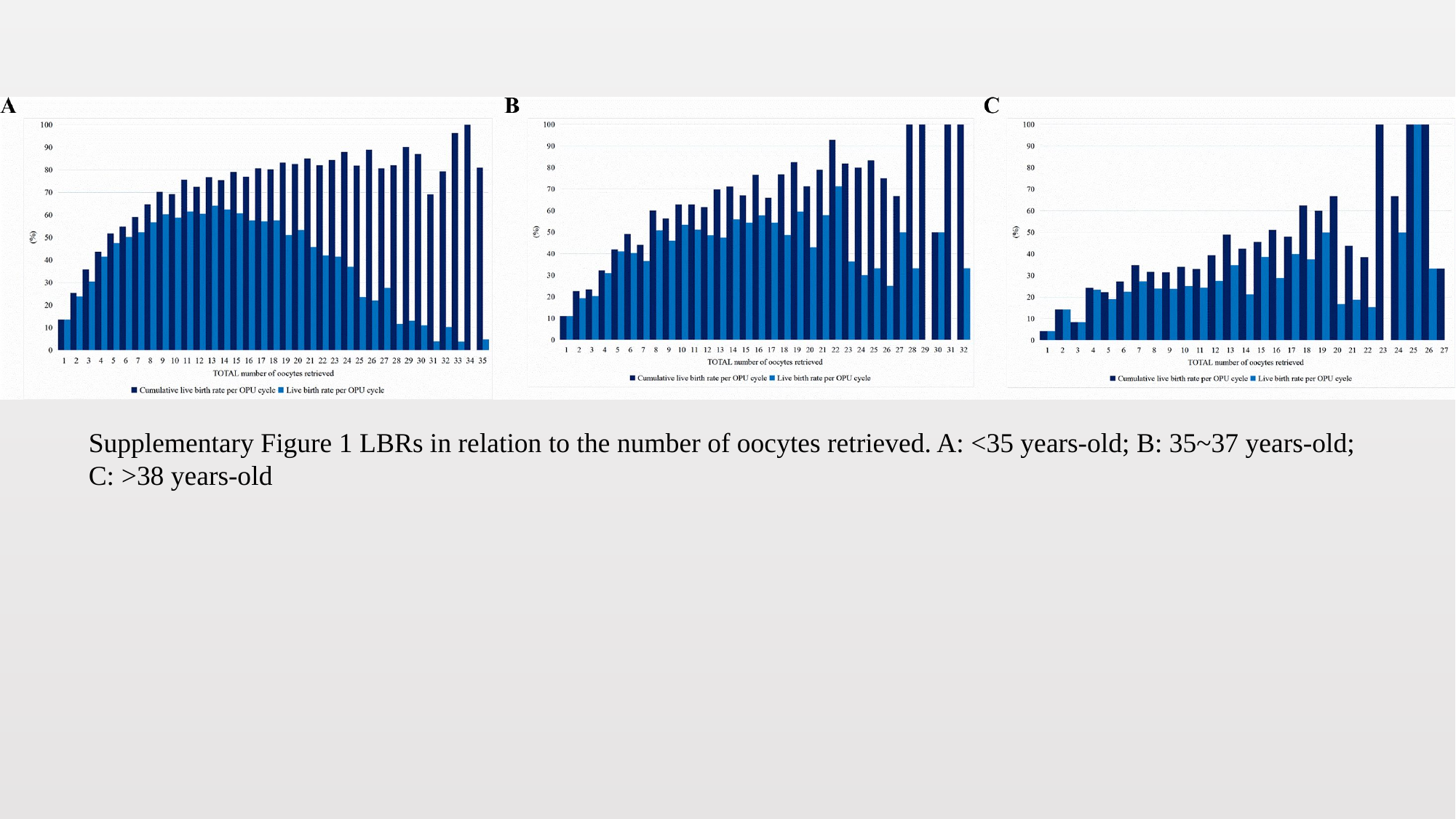

Supplementary Figure 1 LBRs in relation to the number of oocytes retrieved. A: <35 years-old; B: 35~37 years-old; C: >38 years-old

## Slide 2
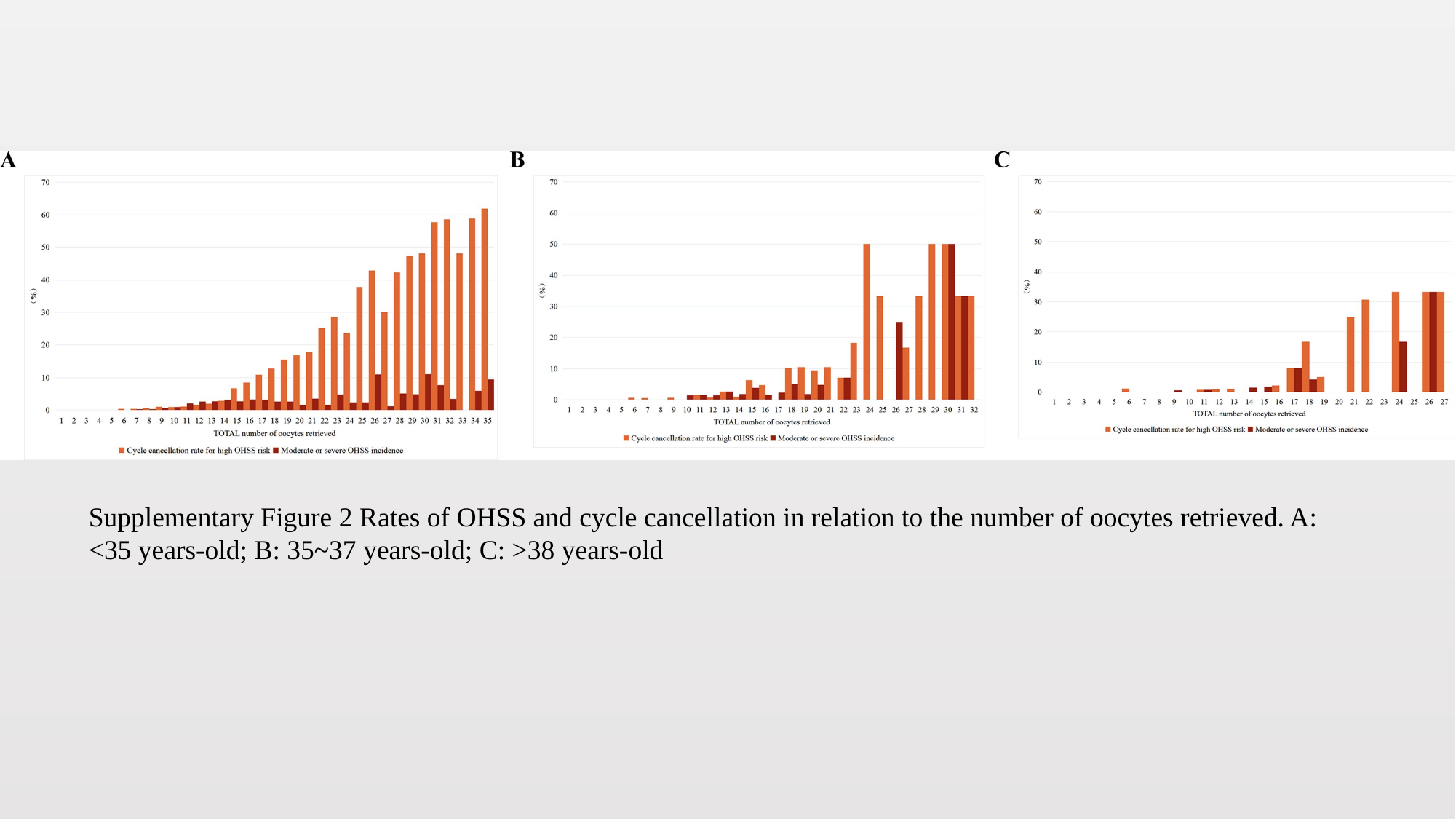

Supplementary Figure 2 Rates of OHSS and cycle cancellation in relation to the number of oocytes retrieved. A: <35 years-old; B: 35~37 years-old; C: >38 years-old
